# Using ratings of perceived difficulty for balance exercise prescription and intensity progression

**DOI:** 10.3389/fresc.2026.1715809

**Published:** 2026-02-10

**Authors:** Saud F. Alsubaie, Gregory F. Marchetti, Kathleen H. Sienko, Susan L. Whitney, Joseph M. Furman, Patrick J. Sparto

**Affiliations:** 1 Department of Health and Rehabilitation Sciences, Prince Sattam Bin Abdulaziz University, Alkharj, Saudi Arabia; 2 Department of Physical Therapy, Duquesne University, Pittsburgh, PA, United States; 3 Department of Mechanical Engineering, University of Michigan, Ann Arbor, MI, United States; 4 Department of Physical Therapy, University of Pittsburgh, Pittsburgh, PA, United States; 5 Department of Otolaryngology, University of Pittsburgh, Pittsburgh, PA, United States

**Keywords:** balance, exercise prescription, intensity progression, perceptual, physical therapy, rehabilitation

## Abstract

**Background:**

Balance is a fundamental component of daily activities and plays a critical role in preventing falls. Balance can be influenced by a variety of factors, including age-related physiological changes, making it important to consider age when assessing balance performance. However, an empirical basis for estimating the difficulty of balance exercises has yet to be developed. The primary aim of this study was to determine the effect of age and different balance exercise conditions on difficulty of exercises as determined by self-reported perceived difficulty, and to show that Rating of Perceived Difficulty (RPD) can serve as a practical measure of difficulty for guiding balance exercise prescription and progression.

**Methods:**

Sixty-two healthy adults between the ages of 20 and 85 years with a mean age of 55 ± 20 years (50% female) participated in this cross-sectional study. Subjects performed four 30 s trials of 24 static standing balance exercises, and the average of these four trials was used for analysis. For each exercise, subjects’ ratings of perceived difficulty (RPD) were recorded using a 0–10 scale.

**Results:**

A significant increase in RPD across all balance exercises occurred as age increased (*p* < 0.02). From the youngest age group to the oldest, RPD increased by more than 100%. Ratings of perceived difficulty increased on a foam surface (110%), eyes closed (43%), semi-tandem stance (150%), and head movement (50%) compared with a firm surface, eyes open, feet apart, and head held still, respectively (*p* < 0.01).

**Conclusion:**

RPD measurements across a range of standing balance exercises can be used as a measure of difficulty and a practical tool for prescribing and progressing balance exercises in rehabilitation programs.

## Introduction

Balance rehabilitation is commonly used to improve balance,mobility and reduce risk of falls especially in older adults and people with vestibular disorders (1–4). Balance exercises can be performed in a multitude of ways by combining various modifying factors, such as the use of different surfaces, visual feedback, stance, and head movement conditions (5, 6). A critical component of any exercise prescription is specifying the intensity of the exercise. A systematic review of balance intervention studies found that there was no description of the difficulty of balance exercises (7). Typically, physical therapists progress the difficulty of balance exercises based on their clinical experience by decreasing proprioceptive information (e. g., standing on foam), modifying visual information (e. g., closing eyes), and/or changing the base of support (e. g., standing in semi-tandem stance) (5, 8). Klatt et al. developed a conceptual framework for progressing balance exercises based on exercise difficulty in individuals with vestibular disorders. The framework was developed mainly based on clinical experience of a multidisciplinary team, including physical therapists and engineers, and included feedback from individuals with vestibular disorders (5). However, an empirical basis for grading the difficulty of these exercises is generally lacking, which may limit the optimal prescription and progression of balance exercises.

For aerobic and resistance exercises, there are very well-defined recommendations for how to determine the initial prescription for exercise intensity as well as how to progress the intensity, based on percent of heart rate reserve or maximum weight lifted (9). Furthermore, the rating of perceived exertion for aerobic and resistance exercises was developed to assist in determining how trainees perceive the intensity of activity in cases where the percent of heart rate reserve or the maximum weight that can be lifted cannot be easily measured (10–12). During training programs, rating of perceived exertion scales help to monitor the intensity of the activity and provide healthcare providers with feedback of how hard their clients' feel like they are exercising as well as if their clients are ready to progress to the next level of intensity.

Several groups have attempted to develop a way to grade the difficulty of balance exercises using postural sway measures, observing the amount of sway visually, or recording verbal and nonverbal responses (13–15). However, many clinics do not have the capability to record sway and interpret its results. Furthermore, visual observation is an imprecise tool for measuring postural sway and determining balance exercise difficulty, and evidence of inter-rater reliability has not been established. Without an appropriate balance difficulty measures, clinicians may face challenges in selecting balance exercises appropriate to an individual's ability and progressing them, and may lead to huge variation in which exercises are chosen and progressed across age groups. Ratings of perceived difficulty (RPD) provide a practical and clinically feasible approach that reflects the individual's perceived difficulty of balance tasks. Therefore, the use of RPD scales may be beneficial for determining the difficulty of balance exercises and for establishing a hierarchical classification for exercise difficulty progression. In a recent study, Alsubaie et al. (16) examined the concurrent validity and test-retest reliability of a RPD scale for grading the difficulty of balance exercises and found that the RPD scale was significantly associated with inertial-based measures of body sway. Thus, the use of the RPD scale can provide a simple, affordable, and low technology solution for comparing the difficulty of balance exercises in clinical settings. In addition, this measure may assist clinicians in selecting the most appropriate balance exercises and help in progressing them, potentially improving training effectiveness and reducing fall risk.

Aging is associated with age-related physiological changes that can affect postural control, making it important to consider age when assessing balance performance. Therefore, the present study was designed to determine the effect of age and exercise conditions (surface type, visual input, stance type, and head movement) on RPD. A secondary purpose was to develop a tool for sequencing the relative difficulty of common static standing balance exercises. The proposed sequence of exercise progression aims to guide clinicians and researchers in designing and progressing treatment plans.

## Methods

### Participants

This cross-sectional study included participants who were able to participate independently in activities of daily living. Study participants were distributed into four groups based on age (17–20): 1 (18–44y), 2 (45–59y), 3 (60–74y), and 4 (75–85y). Subjects were excluded if they were unable to stand for 3 min without rest, had two or more falls in the previous year, or had neurological or musculoskeletal disorders affecting balance (self-reported). The study was conducted at the University of Pittsburgh. This study was approved by the Institutional Review Board at the University of Pittsburgh. All subjects provided written informed consent prior to participating in the study. The sample size was calculated using G Power software (version 3.1.9.7) (21) based on models that tested for the main effect of age and main effects of the experimental conditions. To determine the most conservative effect size for differences in postural sway between young and older subjects performing static balance exercises, a pilot study was conducted prior to the main study, which yielded an ANOVA effect size f of 0.2 (which is equivalent to Cohen d of 0.4) for the repeated measures factor of head movement, of which there were 3 levels. In addition, a previous study examined the differences in postural sway between young and older subjects performing static balance exercises (standing with eyes open or open in the dark, on fixed or movable surfaces) reported an ANOVA effect size f of 0.6 (22). The most conservative effect size (0.2) was therefore used. As a result, we found that sample size in each group should be 11 participants, assuming an alpha level of 0.05, a statistical power of 0.80, using an ANOVA repeated measures model with 4 groups and 3 measurements for each group.

### Experimental procedure

Participants were tested during two experimental visits, one week apart. During each experimental visit, participants performed two sets of 24 randomized static standing balance exercises. The 24 exercises were a full-factorial design of the following different conditions: surface (firm and foam); vision (eyes open and eyes closed); stance (feet apart and semi-tandem); and head movements [head still, yaw (turning the head left and right), and pitch (nodding the head forward and backward)] as shown in Table 1. During head movements, participants moved their heads at a frequency of 1 Hz by synchronizing their movements with a metronome beat. During each set, each exercise was performed once in a random order generated by a software.

**Table 1:** Description of the 24 balance exercises performed in the study.

Exercise number	Surface	Visual input	Base of support	Head movement
1	Firm	Eyes open	Feet apart	Head still
2	Firm	Eyes open	Feet apart	Yaw
3	Firm	Eyes open	Feet apart	Pitch
4	Firm	Eyes open	Semi-tandem	Head still
5	Firm	Eyes open	Semi-tandem	Yaw
6	Firm	Eyes open	Semi-tandem	Pitch
7	Firm	Eyes closed	Feet apart	Head still
8	Firm	Eyes closed	Feet apart	Yaw
9	Firm	Eyes closed	Feet apart	Pitch
10	Firm	Eyes closed	Semi-tandem	Head still
11	Firm	Eyes closed	Semi-tandem	Yaw
12	Firm	Eyes closed	Semi-tandem	Pitch
13	Foam	Eyes open	Feet apart	Head still
14	Foam	Eyes open	Feet apart	Yaw
15	Foam	Eyes open	Feet apart	Pitch
16	Foam	Eyes open	Semi-tandem	Head still
17	Foam	Eyes open	Semi-tandem	Yaw
18	Foam	Eyes open	Semi-tandem	Pitch
19	Foam	Eyes closed	Feet apart	Head still
20	Foam	Eyes closed	Feet apart	Yaw
21	Foam	Eyes closed	Feet apart	Pitch
22	Foam	Eyes closed	Semi-tandem	Head still
23	Foam	Eyes closed	Semi-tandem	Yaw
24	Foam	Eyes closed	Semi-tandem	Pitch

Subjects were asked to stand as still as possible without wearing shoes with their arms at their side. Subjects wore a safety harness and were guarded by a physical therapist to ensure participants' safety. The safety harness was not fully tightened, allowing participants the necessary freedom of movement. There was a seated rest break of 1 min every three exercises to avoid fatigue. The independent variables included age groups, surface, stance, visual, and head conditions, while the dependent variables were postural sway measures and RPD scores.

### Outcome measures

Subjects were asked to provide a rating of perceived difficulty (RPD) of each exercise using a valid and reliable scale that ranged from 0 to 10 with verbal anchors developed by Alsubaie et al. (16), where 0 indicates extremely easy and 10 indicates extremely hard. During the experiment, the RPD scale was placed visibly on the side wall so that subjects could refer to it as needed. Participants who normally wore glasses were instructed to wear them during the experiment.

Supplemental sway data was recorded using an inertial sensor (IMU, Xsense Technologies B.V., Enschede, The Netherlands) was placed on the subject's lower back at the level of the iliac crest (L4) to measure the root-mean-square (RMS) of trunk angular displacement and velocity in the pitch and roll planes, and linear acceleration in anterior/posterior (AP) and medial/lateral (ML) directions. Postural sway measures were recorded for 35 s in each trial. Five seconds were removed from the beginning of each trial to minimize potential effects of the subject's initial establishment of balance (23, 24). The data were recorded at a sampling rate of 100 Hz and low-pass filtered using a second order Butterworth filter with a cut-off frequency of 3 Hz (25, 26). This data can be used by researchers to compare with other studies that recorded postural sway (Supplemental Digital Content 1, Table 2).

**Table 2:** Mean and SD of the rating of perceived difficulty scores for each level of the independent variables. The test statistic and *p*-value are provided, where the effect of Age was tested using the Kruskal–Wallis test, the effect of Surface, Vision, and Stance were assessed using the Wilcoxon signed ranks test, and the effect of Head Movement was tested using the Friedman test.

Variables	Level	Rating of perceived difficulty (0 lowest, 10 highest)		
		Mean	SD	Test *p*-value
Age	18–44y	2.43	0.86	*H* = 27.3 *p* < 0.001
	45–59y	3.31	1.05	
	60–74y	3.80	0.97	
	75–85y	4.98	1.25	
Surface	Firm	2.32	1.32	*Z* = 6.85 *p* < 0.001
	Foam	4.87	1.54	
Vision	Eyes open	2.96	1.35	*Z* = 6.85 *p* < 0.001
	Eyes closed	4.22	1.45	
Stance	Feet apart	2.05	1.20	*Z* = 6.85 *p* < 0.001
	Semi-tandem	5.13	1.73	
Head Movement	Head still	2.68	1.34	*H* = 93.3 *p* < 0.001
	Yaw	4.07	1.45	
	Pitch	4.02	1.45	

### Statistical analyses

For the ordinal RPD data, the Kruskal–Wallis test was used for comparison of Age groups and Dunn's procedure was used for pairwise comparisons using a Bonferroni correction. The Wilcoxon signed-rank test was used for comparison of Stance, Vision, and Surface conditions. The Friedman test was used for comparison of the Head Movement condition, followed by Wilcoxon signed-rank tests for pairwise comparisons with a Bonferroni correction for multiple comparisons (27). The mean value of all four trials (two trials per visit and two visits) of the rating of perceived difficulty was used. The significance level was α = 0.05.

A hierarchical cluster analysis (HCA) was used to categorize the exercises into five clusters (categories) (very easy, easy, moderate, hard, and very hard) to establish a basis for exercise progression. The HCA was conducted using the RPD scale (28).

## Results

### Subjects

Seventy-two participants were initially screened and 10 were excluded because they did not meet the inclusion criteria. The included 62 participants (31 females and 31 males) were between the ages of 18 and 85 years old and had a mean age of 55 ± 20 years. Study participants were distributed into four groups based on age: 1 (18–44y; *n* = 17), 2 (45–59y; *n* = 15), 3 (60–74y; *n* = 15), and 4 (75–85y; *n* = 15).

### Rating of perceived difficulty

The mean scores of the RPD scale are displayed in Table 2. There was a significant difference between the Age groups (*H* = 27.3, *p* < 0.001), with an average 105% increase in RPD from the youngest to the oldest group. The pairwise comparisons showed significant differences between the 18–44y group and 60–74y and 75–85y groups, and between the 45–59y and 75–85y groups. There was a significant difference between the RPD scores due to the effect of Surface (*Z* = 6.85, *p* < 0.001), Vision (*Z* = 6.85, *p* < 0.001), and Stance (*Z* = 6.85, *p* < 0.001), with increased ratings on foam compared with firm (103% increase), eyes closed vs. eyes open (43% increase), and semi-tandem stance related to feet apart (150% increase). The mean RPD score also was significantly different between the different types of head movement (H = 93.3, *p* < 0.001). The *post-hoc* analysis revealed statistically significant differences between the head still, and yaw and pitch head movements (50% increase). Some participants had difficulty completing some of the balance tasks. Most of the participants particularly older adults, could not complete performing exercises #23 and #24.

### Hierarchical cluster analysis

A hierarchical cluster analysis (HCA) was performed to categorize the exercises across all age groups into five clusters based on rating of perceived difficulty in order to inform a quantitative basis for exercise progression. Table 3 is organized by rank ordering the exercises by RPD from smallest to largest rating. The perceived easiest exercises in cluster 1 were dominated by the feet apart stance and firm surface conditions. The most difficult clusters 4 and 5 primarily involved semi-tandem stance and foam surface conditions. In order to use the relative difficulty of the RPD and sway velocity measures in clinical practice, sortable tables of the 24 exercises can be used (see Spreadsheet, Supplemental Digital Content 2 for the sortable tables).

**Table 3:** Exercise clusters based on rating of perceived difficulty (RPD) scores. Table is organized by rank ordering the exercises by RPD rating from smallest to largest.

Exercise number	Surface	Vision	Stance	Head movement	Mean RPD (cluster)
1	Firm	EO	FA	Head still	0.44 (1)
7	Firm	EC	FA	Head still	0.82 (1)
2	Firm	EO	FA	Yaw	1.04 (1)
3	Firm	EO	FA	Pitch	1.05 (1)
8	Firm	EC	FA	Yaw	1.39 (1)
9	Firm	EC	FA	Pitch	1.56 (1)
13	Foam	EO	FA	Head still	1.80 (1)
4	Firm	EO	ST	Head still	2.30 (2)
19	Foam	EC	FA	Head still	2.62 (2)
14	Foam	EO	FA	Yaw	2.66 (2)
15	Foam	EO	FA	Pitch	2.95 (2)
10	Firm	EC	ST	Head still	3.11 (2)
6	Firm	EO	ST	Pitch	3.22 (2)
5	Firm	EO	ST	Yaw	3.42 (2)
20	Foam	EC	FA	Yaw	3.96 (3)
16	Foam	EO	ST	Head still	4.12 (3)
21	Foam	EC	FA	Pitch	4.32 (3)
12	Firm	EC	ST	Pitch	4.50 (3)
11	Firm	EC	ST	Yaw	4.92 (3)
18	Foam	EO	ST	Pitch	6.17 (4)
22	Foam	EC	ST	Head still	6.27 (4)
17	Foam	EO	ST	Yaw	6.38 (4)
24	Foam	EC	ST	Pitch	8.38 (5)
23	Foam	EC	ST	Yaw	8.74 (5)

EO, eyes open; EC, eyes closed; FA, feet apart; ST, semi-tandem; RMS, root mean square.

## Discussion

The present study was designed to determine the effect of age and exercise conditions on ratings of perceived difficulty as a measure of balance exercise intensity. The results of this study showed that ratings of perceived difficulty increased as age increased from the youngest to the oldest age group. In addition, ratings of perceived difficulty were significantly higher when decreasing proprioception information by standing on foam, lack of visual information by closing eyes, narrowing base of support by standing in semi-tandem stance, and altering vestibular function through head movements.

The results of this study, which relied on rating of perceived difficulty to assess balance exercises difficulty, are consistent with the results of other studies that used postural sway measures to quantify balance exercises, which showed that body postural sway increase with aging (17–19, 29–31). The effect of aging on balance performance may be explained by age-related physiological changes, including declines in vestibular and proprioceptive function, muscle strength, and central processing speed. These age-related changes may cause older adults to depend more on compensatory strategies, which can reduce postural stability. As a result, when younger and older individuals report the same level of perceived difficulty for the same balance task, we should consider the underlying physiological demands and balance control strategies for both age groups. Older adults may need wider limits of stability and require greater neuromuscular effort to achieve comparable task performance. Therefore, self-reported difficulty should be interpreted with consideration of age-related factors when guiding balance training progression. Similarly, postural sway has been shown to increase with alteration of sensory information. An increase in body sway was found with alteration of sensory information through decreasing proprioception information by standing on foam (17, 30, 32), removing visual cues by closing eyes (17, 18, 30, 32, 33), and narrowing the base of the support by standing in semi-tandem stance (18). Furthermore, previous research has demonstrated that ratings of perceived difficulty are significantly associated with measures of postural sway, indicating that ratings of perceived difficulty may serve as an independent measure of balance (16). These results agree with the results of our study, where participants gave higher RPD scores for the more challenging conditions, showing that they perceived these exercises as more difficult.

The study findings in terms of progressing balance exercises, generally reflect the conceptual framework of progression for static standing balance exercises as proposed by Klatt et al. (5). However, in the conceptual framework, it was suggested that semi-tandem stance with eyes open was less challenging than feet apart with eyes closed, while holding other exercise conditions constant. Alternatively, our subjects perceived semi-tandem stance with eyes open to be more challenging than feet apart with eyes closed. Additionally, Klatt et al. reported that exercises with head movements presented a greater challenge for individuals with vestibular disorders compared with modifying the stance. However, we found that exercises with head movements with feet apart produced less sway velocity compared with semi-tandem stance with the head still.

The importance of self-reported difficulty in balance training should be considered, as it reflects participants' perceived difficulty of balance tasks, an area that may not be fully captured by objective measures of postural sway. Previous research has shown that appropriately matching task difficulty to an individual's abilities can enhance self-efficacy and positive performance expectations, both of which are key components of motor learning. According to the Challenge Point Theory, optimal motor learning occurs when task difficulty is appropriately match the learner's skill, being neither too easy nor too difficult (34). This condition may be achieved by setting task difficulty based on individual's self-reported ratings of perceived difficulty. Additionally, setting task difficulty based on individual's self-reported difficulty enhance the sense of control and choice in individuals, which in turn enhance self-efficacy and positive performance expectations leading to better motor performance and learning (35). In this context, ratings of perceived difficulty may provide a practical and individualized approach to guiding balance training progression.

The quantification rating of perceived difficulty establishes a foundation for estimating intensity of standing balance exercises which can be used in rehabilitation settings. Clinicians can take advantage of the measured exercise intensity to select an appropriate exercise for their clients based on their age. Using the spreadsheet included (see Spreadsheet, Supplemental Digital Content 2), one can sort all of the exercises based on the entire sample or based on a specific age group. If a therapist would like to target a specific sensory condition (e.g., surface or vision), the data can be sorted to select the progression of exercises for only those exercise categories. Clinicians can use the developed rating of perceived difficulty scale to estimate the intensity of each exercise in situations when sway cannot be measured.

This study has some limitations that should be noted. First, participants were not specifically screened for comorbidities, and information on neurological or musculoskeletal conditions that may influence balance was based solely on self-report. However, all participants were tested prior to inclusion by standing for three minutes to ensure they could maintain balance before entering the study. Another limitation related to the length of experimental sessions. Experimental sessions lasted on average approximately more than one hour and a half, which could potentially lead to fatigue, particularly in older adults; however, short seated rest breaks of one minute were provided after every three exercises to minimize fatigue. Additionally, testing conditions were randomized across sessions and visits to reduce potential order effects related to practice or fatigue. One limitation of this study is that vestibular disorders were not specifically assessed and were not included among the exclusion criteria. Therefore, participants with undiagnosed vestibular disorders may have been included. Since vestibular disorders can greatly affect balance, the results of this study cannot be generalized to populations with vestibular-related balance issues and should be interpreted with caution when considering individuals with vestibular disorders. Another limitation is the use of a safety harness during testing. While the harness was necessary for safety, it may have provided participants with a sense of reassurance, reduced fear of falling, influenced their performance, and affected their perceived difficulty of the balance tests. Therefore, the results could be different if no harness was used.

## Conclusions

An empirical basis for estimating intensity of standing balance exercises based on self-reported rating of perceived difficulty was established. The increase in magnitude of ratings of perceived difficulty measures varied across the manipulation of different sensory inputs and biomechanical constraints. The results of the study show that RPD can serve as a practical measure of intensity of standing balance exercises. Additionally, RPD and can be used as a guide for prescription and progression of standing balance exercises in rehabilitation programs.

## Data Availability

The raw data supporting the conclusions of this article will be made available by the authors, without undue reservation.

## References

[1] Horak FB Jones-Rycewicz C Black FO Shumway-Cook A. Effects of vestibular rehabilitation on dizziness and imbalance. Otolaryngol Head Neck Surg. (1992) 106(2):175–80. 10.1177/019459989210600220 1738550

[2] Hillier SL McDonnell M. Vestibular rehabilitation for unilateral peripheral vestibular dysfunction. Cochrane Database Syst Rev. (2011) (2):CD005397. 10.1002/14651858.CD005397.pub3 21328277

[3] Barnett A Smith B Lord SR Williams M Baumand A. Community-based group exercise improves balance and reduces falls in at-risk older people: a randomised controlled trial. Age Ageing. (2003) 32(4):407–14. 10.1093/ageing/32.4.407 12851185

[4] Howe TE Rochester L Neil F Skelton DA Ballinger C. Exercise for improving balance in older people. Cochrane Database Syst Rev. (2011) 2011(11):CD004963. 10.1002/14651858.CD004963.pub3 22071817 PMC11493176

[5] Klatt BN Carender WJ Lin CC Alsubaie SF Kinnaird CR Sienko KH A conceptual framework for the progression of balance exercises in persons with balance and vestibular disorders. Physical Med Rehabil Int. (2015) 2(4):1044. PMC4968039 27489886

[6] Alsalaheen BA Whitney SL Mucha A Morris LO Furman JM Sparto PJ. Exercise prescription patterns in patients treated with vestibular rehabilitation after concussion. Physiother Res Int. (2013) 18(2):100–8. 10.1002/pri.1532 22786783 PMC4894842

[7] Farlie MK Robins L Keating JL Molloy E Haines TP. Intensity of challenge to the balance system is not reported in the prescription of balance exercises in randomised trials: a systematic review. J Physiother. (2013) 59(4):227–35. 10.1016/S1836-9553(13)70199-1 24287216

[8] Herdman SJ Clendaniel RA. Vestibular Rehabilitation. Philadelphia, PA: F. A. Davis Company (2014).

[9] Pescatello LS, American College of Sports Medicine. ACSM’s Guidelines for Exercise Testing and Prescription. 9th ed. Philadelphia, PA: Wolters Kluwer/Lippincott Williams & Wilkins Health (2014). xxiv. p. 456.

[10] Robertson RJ Goss FL Rutkowski J Lenz B Dixon C Timmer J Concurrent validation of the OMNI perceived exertion scale for resistance exercise. Med Sci Sports Exercise. (2003) 35(2):333–41. 10.1249/01.MSS.0000048831.15016.2A 12569225

[11] Utter AC Robertson RJ Green JM Suminski RR McAnulty SR Nieman DC. Validation of the adult OMNI scale of perceived exertion for walking/running exercise. Med Sci Sports Exercise. (2004) 36(10):1776–80. 10.1249/01.MSS.0000142310.97274.94 15595300

[12] Robertson RJ Goss FL Dube J Rutkowski J Dupain M Brennan C Validation of the adult OMNI scale of perceived exertion for cycle ergometer exercise. Med Sci Sports Exercise. (2004) 36(1):102–8. 10.1249/01.MSS.0000106169.35222.8B 14707775

[13] Muehlbauer T Roth R Bopp M Granacher U. An exercise sequence for progression in balance training. J Strength Cond Res. (2012) 26(2):568–74. 10.1519/JSC.0b013e318225f3c4 22067238

[14] Farlie MK Molloy E Keating JL Haines TP. Clinical markers of the intensity of balance challenge: observational study of older adult responses to balance tasks. Phys Ther. (2016) 96(3):313–23. 10.2522/ptj.20140524 26183587

[15] Alsubaie SF Whitney SL Furman JM Marchetti GF Sienko KH Sparto PJ. Reliability of postural sway measures of standing balance tasks. J Appl Biomech. (2018) 35:1–23. 10.1123/jab.2017-0322 PMC6328343 29989455

[16] Alsubaie SF Whitney SL Furman JM Marchetti GF Sienko KH Klatt BN Reliability and validity of ratings of perceived difficulty during performance of static standing balance exercises. Phys Ther. (2019) 99(10):1381–93. 10.1093/ptj/pzz091 31309968 PMC6821262

[17] Gill J Allum JHJ Carpenter MG Held-Ziolkowska M Adkin AL Honegger F Trunk sway measures of postural stability during clinical balance tests: effects of age. J Gerontol A Biol Sci Med Sci. (2001) 56(7):M438–47. 10.1093/gerona/56.7.m438 11445603

[18] Era P Sainio P Koskinen S Haavisto P Vaara M Aromaa A. Postural balance in a random sample of 7,979 subjects aged 30 years and over. Gerontology. (2006) 52(4):204–13. 10.1159/000093652 16849863

[19] Baloh RW Jacobson KM Enrietto JA Corona S Honrubia V. Balance disorders in older persons: quantification with posturography. Otolaryngol Head Neck Surg. (1998) 119(1):89–92. 10.1016/S0194-5998(98)70177-9 9674519

[20] Rosenhall U Rubin W. Degenerative changes in the human vestibular sensory epithelia. Acta Otolaryngol. (1975) 79(1–2):67–80. 10.3109/00016487509124657 167544

[21] Faul F Erdfelder E Lang AG Buchner A. G*Power 3: a flexible statistical power analysis program for the social, behavioral, and biomedical sciences. Behav Res Methods. (2007) 39(2):175–91. 10.3758/BF03193146 17695343

[22] Lin CC. The Effect of Vibrotactile Feedback on Healthy People and People with Vestibular Disorders during Dual-task Conditions. (2014):65–74. 13 January 2014. (Accessed 10 July, 2014).

[23] O’Sullivan M Blake C Cunningham C Boyle G Finucane C. Correlation of accelerometry with clinical balance tests in older fallers and non-fallers. Age Ageing. (2009) 38(3):308–13. 10.1093/ageing/afp009 19252205

[24] Rine RM Schubert MC Whitney SL Roberts D Redfern MS Musolino MC Vestibular function assessment using the NIH Toolbox. Neurology. (2013) 80(11 Suppl 3):S25–31. 10.1212/WNL.0b013e3182872c6a 23479540 PMC3662339

[25] Dozza M Chiari L Horak FB. Audio-biofeedback improves balance in patients with bilateral vestibular loss. Arch Phys Med Rehabil. (2005) 86(7):1401–3. 10.1016/j.apmr.2004.12.036 16003671

[26] Dozza M Horak FB Chiari L. Auditory biofeedback substitutes for loss of sensory information in maintaining stance. Exp Brain Res. (2007) 178(1):37–48. 10.1007/s00221-006-0709-y 17021893

[27] Dunn OJ. Multiple comparisons among means. J Am Stat Assoc. (1961) 56(293):13. 10.1080/01621459.1961.10482090

[28] Zhang Z Murtagh F Van Poucke S Lin S Lan P. Hierarchical cluster analysis in clinical research with heterogeneous study population: highlighting its visualization with R. Ann Transl Med. (2017) 5(4):75. 10.21037/atm.2017.02.05 28275620 PMC5337204

[29] Liaw MY Chen CL Pei YC Leong CP Lau YC. Comparison of the static and dynamic balance performance in young, middle-aged, and elderly healthy people. Chang Gung Med J. (2009) 32(3):297–304. 19527609

[30] Abrahamova D Hlavacka F. Age-related changes of human balance during quiet stance. Physiol Res. (2008) 57(6):957–64. 10.33549/physiolres.931238 18052683

[31] Alsubaie SF. The postural stability measures most related to aging, physical performance, and cognitive function in healthy adults. Biomed Res Int. (2020) 2020:5301534. 10.1155/2020/5301534 32908898 PMC7463407

[32] Cohen H Heaton LG Congdon SL Jenkins HA. Changes in sensory organization test scores with age. Age Ageing. (1996) 25(1):39–44. 10.1093/ageing/25.1.39 8670527

[33] Hytonen M Pyykko I Aalto H Starck J. Postural control and age. Acta Otolaryngol. (1993) 113(2):119–22. 10.3109/00016489309135778 8475724

[34] Guadagnoli MA Lee TD. Challenge point: a framework for conceptualizing the effects of various practice conditions in motor learning. J Mot Behav. (2004) 36(2):212–24. 10.3200/JMBR.36.2.212-224 15130871

[35] Wulf G Lewthwaite R. Optimizing performance through intrinsic motivation and attention for learning: the OPTIMAL theory of motor learning. Psychon Bull Rev. (2016) 23(5):1382–414. 10.3758/s13423-015-0999-9 26833314

